# Transcriptome Analysis Reveals the Immune Infiltration Profiles in Cervical Cancer and Identifies KRT23 as an Immunotherapeutic Target

**DOI:** 10.3389/fonc.2022.779356

**Published:** 2022-06-24

**Authors:** Xia Li, Yan Cheng, Yanmei Cheng, Huirong Shi

**Affiliations:** ^1^ Gynecological Oncology Radiotherapy Ward, The First Affiliated Hospital of Zhengzhou University, Zhengzhou, China; ^2^ Department of Gynecology and Obstetrics, The First Affiliated Hospital of Zhengzhou University, Zhengzhou, China

**Keywords:** cervical cancer, hot and cold tumor, KRT23, prediction model, tumor microenvironment

## Abstract

Cervical cancer (CC) is one of the most common malignancies in women worldwide. Dismal prognosis rates have been associated with conventional therapeutic approaches, emphasizing the need for new strategies. Recently, immunotherapy has been used to treat various types of solid tumors, and different subtypes of the tumor microenvironment (TME) are associated with diverse responses to immunotherapy. Accordingly, understanding the complexity of the TME is pivotal for immunotherapy. Herein, we used two methods, “ssGSEA” and “xCell,” to identify the immune profiles in CC and comprehensively assess the relationship between immune cell infiltration and genomic alterations. We found that more adaptive immune cells were found infiltrated in tumor tissues than in normal tissues, whereas the opposite was true for innate cells. Consensus clustering of CC samples based on the number of immune cells identified four clusters with different survival and immune statuses. Then, we subdivided the above four clusters into “hot” and “cold” tumors, where hot tumors exhibited higher immune infiltration and longer survival time. Enrichment analyses of differentially expressed genes (DEGs) revealed that the number of activated immune signaling pathways was higher in hot tumors than that in cold tumors. Keratin, type I cytoskeletal 23 (KRT23), was upregulated in cold tumors and negatively correlated with immune cell infiltration. *In vitro* experiments, real-time reverse transcription-quantitative polymerase chain reaction, cytometric bead arrays, and ELISA revealed that knockdown of KRT23 expression could promote the secretion of C-C motif chemokine ligand-5 and promote the recruitment of CD8^+^ T cells. We also constructed a model based on DEGs that exhibited a high predictive power for the survival of CC patients. Overall, our study provides deep insights into the immune cell infiltration patterns of CC. Moreover, KRT23 has huge prospects for application as an immunotherapeutic target. Finally, our model demonstrated a good predictive power for the prognosis of CC patients and may guide clinicians during immunotherapy.

## Introduction

Cancer is widely acknowledged to pose the highest clinical, social, and economic burden in terms of cause-specific disability-adjusted life years (1). Cervical cancer (CC) is ranked fourth for incidence and mortality in women worldwide (2). Invasion and metastasis by CC cells are associated with a poor prognosis, representing the most prevalent cause of cancer-associated deaths (3, 4). Current evidence suggests that surgery, chemotherapy, and radiotherapy yield satisfactory efficacy for early-stage and low-risk CC (5–7). However, the reported 5-year survival for metastatic cervical cancer is only 16.5% (8). In addition, side effects caused by chemotherapy and radiotherapy limit their clinical use. Accordingly, the exploration of the biological mechanisms and the development of new therapeutic targets and strategies for CC patients are essential.

In recent years, many emphases have been placed on the crucial role of immunotherapy in CC. Given the high expression of programmed cell death-1 (PD-1) and programmed cell death ligand-1(PD-L1) in advanced CC, an increasing body of evidence suggests that pembrolizumab (a humanized monoclonal anti-PD-1 antibody) yields substantial antitumor activity and exhibits a good biosafety profile in clinical trials during the treatment of recurrent CC or metastatic CC (mCC) (9–11). Even immunotherapy has achieved remarkable efficacy. Accumulated data in recent years have demonstrated that many patients experience minimal or no clinical benefit if provided with identical treatment. This phenomenon has been attributed to the complexity and uniqueness of the tumor microenvironment (TME).

The TME is a complex, plastic, and dynamic system sculpted by tumor cells and other surrounding cells (12, 13). Cells from the innate immune system and adaptive immune system, representing important components of the tumor stroma, can be reprogrammed according to the TME and may be involved in the survival and progression of tumor cells (14, 15). For example, tumor-associated macrophages (TAMs) represent the largest population of infiltrating myeloid cells in most solid tumors (16). It has been established that TAMs display a high degree of functional plasticity when exposed to various microenvironmental conditions and can be classified as “M1-like” (pro-inflammatory and usually antitumor) or “M2-like” (anti-inflammatory and protumor) (17, 18). Accumulating evidence substantiates the critical roles of the TME in promoting tumor progression. However, it remains unclear how the TME affects the efficacy of immunotherapy in CC. It is well-recognized that immunotherapy harnesses or restores the immune system to kill tumor cells, but this process requires the infiltration of immune cells in the tumor site. Many studies have demonstrated that different types of TMEs are associated with diverse degrees of clinical efficacy with immunotherapy. In this regard, a “hot” tumor with sufficient tumor-infiltrating lymphocytes and antigen-presenting cells can robustly respond to immunotherapy. In contrast, a “cold” tumor lacking immune cells, in general, cannot elicit an effective response to immunotherapy (19). Therefore, understanding and distinguishing the unique classes of the TME are useful for predicting and guiding immunotherapy.

Herein, we undertook a comprehensive analysis to explore the infiltration of immune cells in CC using two different methods and constructed a prediction model. We observed that CC patients with greater immune cell infiltration survived longer times. To uncover the underlying mechanisms of immune cell infiltration, we subdivided CC tumors into hot and cold types and ascertained the differentially expressed genes (DEGs) between them. Then, we identified Keratin, type I cytoskeletal 23 (KRT23), as a immunotherapeutic target. In addition, our model exhibited good predictive power for the overall survival (OS) of CC patients.

## Materials and Methods

### Ethics Statement

Primary CC specimens were obtained after surgery and were frozen in the biobank of the First Affiliated Hospital of Zhengzhou University; some specimens have received neoadjuvant therapy. All participants provided written informed consent for their specimens to be used in this study. The study protocol was approved by the Ethics Committee of the First Affiliated Hospital of Zhengzhou University.

### Cell Culture

A human cervical cell line (HeLa) was purchased from the Institute of Biochemistry and Cell Biology of the Chinese Academy of Sciences (Shanghai, China). Cells were cultured in RPMI1640 medium with 5% fetal bovine serum and an atmosphere of 5% CO_2_ in a humidified incubator at 37°C.

### Acquisition and Normalization of Data

Level-2 mRNA sequencing data (fragment per kilobase of transcript per million mapped reads) of CC were downloaded from The Cancer Genome Atlas (TCGA) database (https://portal.gdc.cancer.gov/) and transformed to transcripts per million for further analyses. The clinical data of CC were downloaded from the University of California Santa Cruz Xena (http://xena.ucsc.edu/). The GSE78220 dataset was downloaded from the Gene Expression Omnibus database (https://www.ncbi.nlm.nih.gov/geo/). A dataset of patients with metastatic urothelial cancer treated with anti-PD-L1 agents downloaded from the online website is supplied in the article (http://research-pub.gene.com/IMvigor210CoreBiologies/).

### Estimation of the Immune Profile

The immune profile (i.e., the number and type of immune cells) was estimated by the R packages “ssGSEA” and “xCell” (R Institute for Statistical Computing, Vienna, Austria). For xCell analysis, we selected samples with *p* < 0.05 and only included immune cells for further analyses. The Immune Score, Stromal Score, and tumor purity were calculated by the R package “ESTIMATE.”

### Identification and Functional Annotation of Differentially Expressed Genes

Tumor samples were divided into “cold” and “hot” subtypes. DEGs were calculated by the R package “Limma” and visualized by volcano plots using the R package “ggplot2.” DEGs with log fold change >1 and *p* < 0.05 were selected for annotation using the Kyoto Encyclopedia of Genes and Genomes (KEGG; https://www.genome.jp/) and Gene Ontology (GO; http://geneontology.org/) databases using the R package “clusterprofile.” A protein–protein interaction (PPI) network was constructed using Search Tool for the Retrieval of Interacting Genes/Proteins (STRING; www.string-db.org/) and visualized by Cytoscape v3.6.1 (https://cytoscape.org/).

### Correlation and Survival Analyses

The R package “corrplot” was used to analyze the correlation of immune cells. The correlation of KRT23 and C-X-C motif chemokine ligand 9 (CXCL9) and CXCL10 and C-C motif chemokine ligand 5 (CCL5) in TCGA dataset was analyzed through cbioportal (www.cbioportal.org/). The correlation of KRT23 and immune cells as well as KRT23 expression in the pan-cancer dataset was determined by the online website TIMER (https://cistrome.shinyapps.io/timer/). Correlation analysis in tumor tissues from patients was conducted by Prism7 (GraphPad, San Diego, CA, USA). For survival analyses, samples were divided into four clusters or “hot” and “cold” tumor. The R package “survival” was used to assess the survival difference using the log-rank test.

### Real-Time Reverse Transcription-Quantitative Polymerase Chain Reaction

Total RNA was extracted by TRIzol^®^Reagent according to the manufacturer’s (TaKaRa Biotechnology, Shiga, Japan) instructions, and the concentration was measured using a spectrophotometer (NanoDrop™ 2000; Thermo Fisher, Waltham, MA, USA). RNA (1 µg) was used to reverse DNA using the PrimeScript™ RT Reagent kit (TaKaRa Biotechnology). The primers for KRT23 were constructed by PrimerBank (https://pga.mgh.harvard.edu/primerbank/index.html/) and synthesized by Sangon Biotech (Shanghai, China) (**Supplementary Table S1**). Glyceraldehyde-3-phosphate dehydrogenase was used for data normalization.

### Small Interfering RNA Transfection

Knockdown of KRT23 expression was achieved using the jetPRIME^®^ Transfection Reagent kit (Polyplus-transfection, Illkirch-Graffenstaden, France). HeLa cells (1 × 10^5^) were seeded in six-well plates with RPMI1640 medium. Before transfection, the small interfering RNA (siRNA) of KRT23 was diluted to 20 µM according to the manufacturer’s instructions. Then, 200 µl of transfection buffer and 4 µl of jetPRIME reagents were mixed and incubated for 10 s at room temperature. Subsequently, 50 nM of siRNA was added and incubated for 15 min at room temperature. siRNA efficacy was analyzed by real-time reverse transcription-quantitative polymerase chain reaction (RT-qPCR) after 48 h. The sequence of siRNA synthesized by Gene Pharma (Shanghai, China) is listed in **Supplementary Table S2.**

### Transwell™ Assay

Migration of CD8^+^ T cells was analyzed through the Transwell assay. CD8^+^ T cells (2 × 10^4^) isolated by microbeads from healthy donors were activated with CD3/CD28 beads and seeded in the upper chamber of the Transwell apparatus with serum-free medium (Millipore, Billerica, MA, USA). HeLa cells (2 × 10^4^) were seeded in the lower chamber with RPMI1640 medium. The number of CD8^+^ T cells was calculated using flow cytometry.

### Enzyme-Linked Immunosorbent Assay

Tumor cells were transfected with siRNA for 48 h. Then, supernatants were collected and centrifuged (1,500 rpm, 5 min) to remove debris. The CCL5 concentration was measured by the LEGEND MAX™ Human CCL5 (regulated upon activation normal T cell expressed and secreted factor, RANTES) ELISA kit according to the manufacturer’s (Biolegend, San Diego, CA, USA) instructions. Briefly, standard dilutions and samples were prepared, followed by the addition of 50 μl of Assay Buffer B to each well. Then, 50 μl of the standard or sample was added to the appropriate well, followed by incubation at room temperature for 2 h with agitation at 200 rpm. Then, 100 μl of Human CCL5 Detection Antibody solution was added to each well, followed by 100 μl of Avidin-HRP A solution. Results were read at an optical density of 450 nm.

### Detection of Multiple Chemokines

We used the LEGENDplex^™^ kit (BioLegend) to detect the chemokines secreted by tumor cells. First, 25 µl of assay buffer was added to the standard or sample in each tube. Then, we added 25 µl of mixed beads (A and B) and incubated at room temperature for 2 h with agitation at 500 rpm. Subsequently, we added 25 µl of antibodies to each tube and incubated at room temperature for 1 h with agitation at 500 rpm. Next, we added 25 µl of SA-PE to each tube and washed it with washing buffer. The fluorescence intensity was detected by a flow cytometer and analyzed by LEGENDplex v8.0.

### Statistical Analyses

Statistical analyses were undertaken using Prism 7 (GraphPad) and R 3.6.3. Two-tailed unpaired *t*-tests and the Wilcoxon test were used to compare the difference between the two groups. Spearman’s rank correlation coefficient was used to evaluate the correlation. A *p*-value <0.05 was statistically significant.

## Results

### Infiltration Pattern of Immune Cells in Tumor and Adjacent Normal Tissue

**Figure 1:**
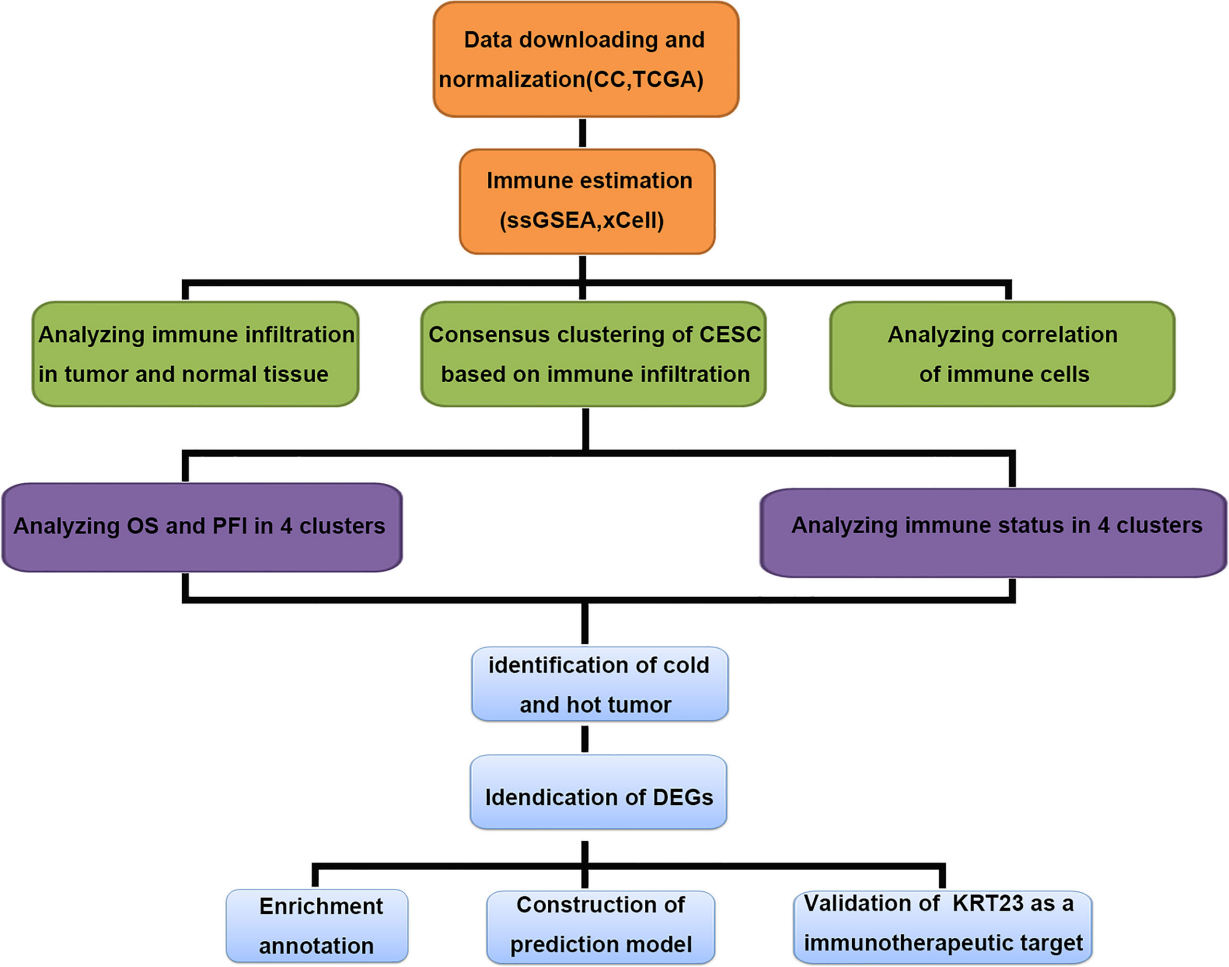
Multiple-step analysis of this study.

**Figure 2:**
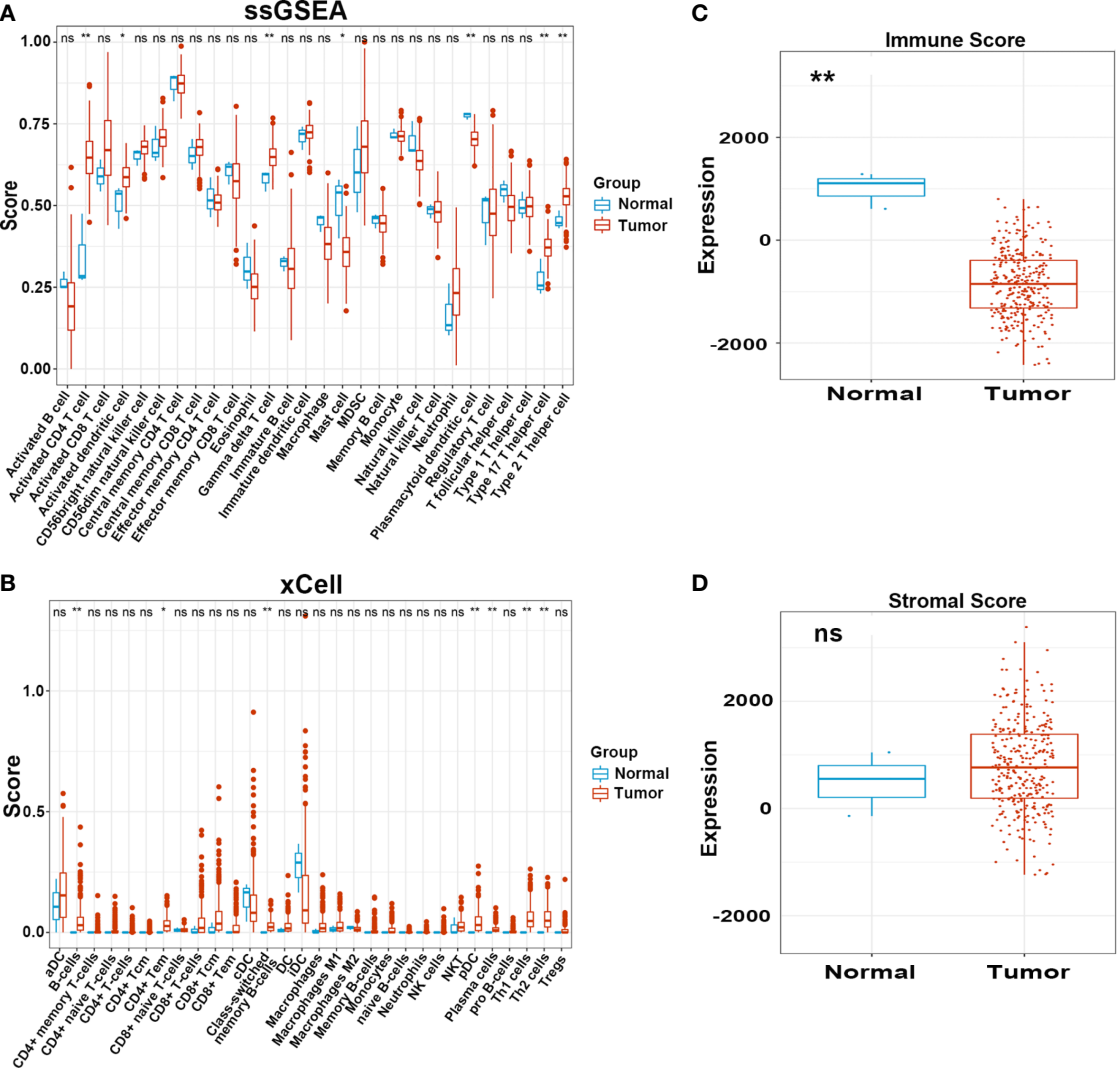
Infiltration pattern of immune cells in adjacent normal and tumor tissues. **(A, B)** Level of immune cells in normal and tumor tissues estimated by ssGSEA and xCell. **(C, D)** Immune and Stromal score in normal and tumor tissues estimated by ESTIMATE. ns, not significant; *p ≤ 0.05, **p ≤ 0.01.

We carried out a multistep analysis to explore the infiltration of immune cells into CC (**Figure 1**). First, we estimated the number of immune cells in each sample between tumor and adjacent normal tissues by Single sample gene set enrichment analysis (ssGSEA) and xCell algorithms. ssGSEA and xCell consistently showed that the number of each cell type that infiltrated into the TME was different, revealing the complexity of the TME. In general, the number of adaptive immune cells, such as activated CD4^+^ T cells, effector memory CD4^+^ T cells, type-17 T-helper (Th17) cells, and Th2 cells, in tumor tissue was higher than that in adjacent normal tissue, which indicated an activated immune response in tumor tissue. The number of CD8^+^ T cells was higher in tumor tissues, but the difference was not statistically significant. Cells from the innate immune system were significantly infiltrated in normal tissues (**Figures 2A, B**). Tumor tissues had a lower Immune Score, but the difference in Stromal Score was not significant (**Figures 2C, D**). We also compared the difference in immune cells in patients who received radiotherapy. After radiotherapy, pro-B cells and Th1 cells accumulated in tumor tissue (**Supplemetary Figures S1A, B**) . Overall, the above results revealed distinct adaptive and innate immune cell infiltration patterns.

### Characterization of Immune Clusters in Cervical Cancer Tissues

**Figure 3:**
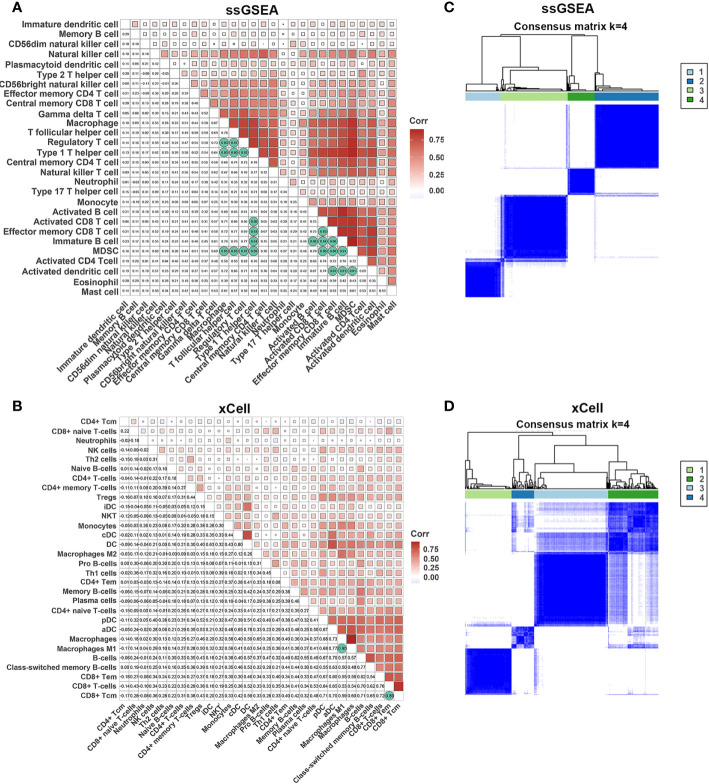
Correlations of immune cells. **(A, B)** Correlation of immune cells in tumor tissues estimated by ssGSEA and xCell. **(C, D)** The heatmap shows the consensus clustering of CC based on levels of immune cells estimated by ssGSEA and xCell.

It is widely acknowledged that an efficient antitumor immune response requires the synergistic action of multiple cells. To explore the relationships between different cell types, we performed a correlation analysis of infiltrating cells in tumor tissues. Most infiltrating cells showed a high correlation with each other, especially activated CD8^+^, CD4^+^ T, dendritic, and B cells. We observed a high correlation between immunosuppressive and immune cells, such as regulatory T cells, myeloid-derived suppressor cells, and M2 macrophages, which suggested that immune suppression was induced by tumor cells after activation of the immune system. The innate immune system cells, such as monocytes, neutrophils, and natural killer cells, exhibited a weak association with other cells, demonstrating a unique antitumor immune process (**Figures 3A, B**).

**Figure 4:**
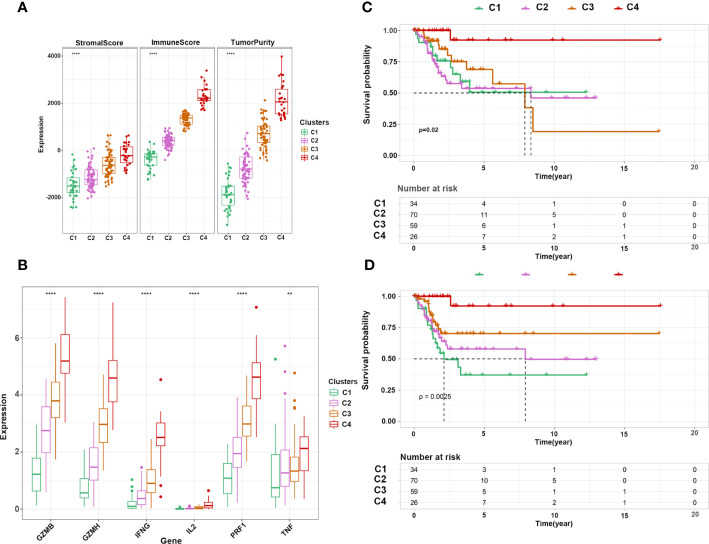
Characterization of immune clusters of CC. **(A)** Expression of immune score, stromal score, and tumor purity in the four subtypes. **(B)** Expression of cytotoxicity-related cytokines in the four subtypes. **(C, D)** Kaplan–Meier curve shows the OS and PFI of 4 clusters. **p ≤ 0.01, ****p ≤ 0.0001.

Next, we performed consensus clustering of all samples based on the proportions of immune cells to identify the subtypes of infiltrating immune cells. The consensus matrix heatmap showed four distinct groups estimated by two methods (**Figures 3C, D**). We observed a gradual increase in immune cell infiltration in tumor tissue from groups 1–4. Groups 1 and 2 demonstrated little infiltration of immune-related cells, group 3 had modest infiltration levels, and group 4 demonstrated high levels of immune cells (**Supplemetary Figures S2A, B**) . Consistently, group 4 had the highest Immune Score (**Supplementary Figures S2C, D**) . To further characterize the clusters of CC cells, we intersected each group obtained from the two methods and denoted them as clusters 1–4 (**Supplementary Figure S3A**) . In accordance with the results stated above, cluster 4 had a high Immune Score (**Figure 4A**). Next, we analyzed the expression of genes involved in the immune response, immune tolerance, and antigen presentation in the four clusters. The expression of immune checkpoint-related genes (*CD276*, *CD274*, *CD40*, *CTLA4*, *HAVCR2*, *LAG3*, *PDCD1*), antigen presentation-related genes (*B2M*, *HLA-B*, *HAL-C*, *HLA-DQA1*, *TAP1*, *TAP2*, *HLA-DQA2*), cytokine-related genes (*GZMB*, *GZMH*, *IFNG*, *PRF1*, *TNF*), and chemokine-related genes (*CCL5*, *CXCL10*, *CXCL13*, *CXCL9*) increased gradually from cluster 1 to cluster 4 (**Figure 4B**; **Supplementary Figures S3B–D**) . Survival analyses revealed that cluster 4 had the longest survival relative to clusters 1, 2, and 3 in terms of OS and progression-free interval (PFI) and have potential trends of a higher percentage of patients with low stages, although there was inconsistency with grade (**Figures 4C, D**; **Supplementary Figures S4A, B**) .

### Survival Status and Signaling Alterations Between Hot Tumors and Cold Tumors

**Figure 5:**
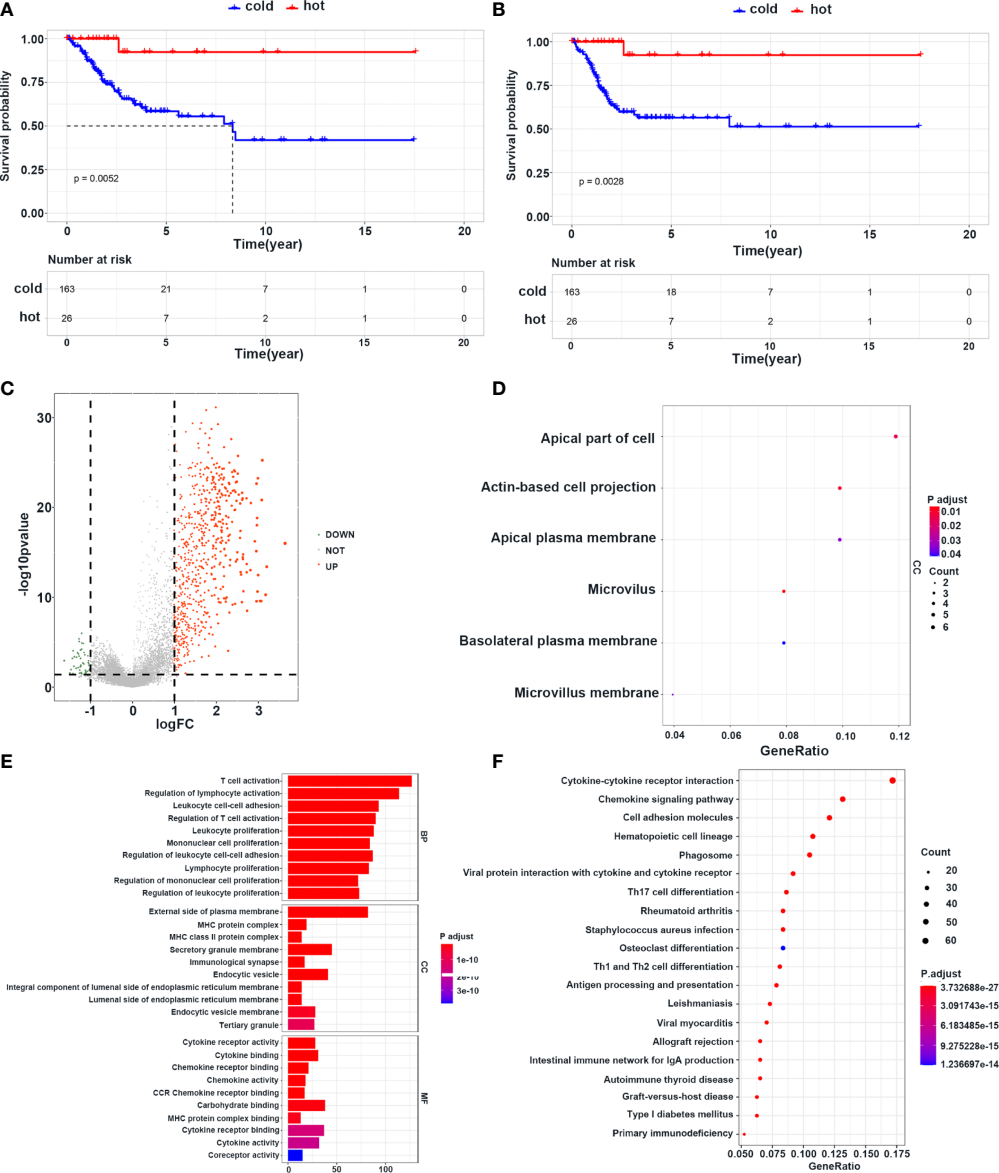
Survival and transcriptome characteristics of cold and hot tumors. **(A, B)** Kaplan–Meier curve shows the OS and PFI of cold and hot tumors. **(C)** Volcano plot shows the difference of gene expression in cold and hot tumors. **(D)** GO enrichment analysis in cold tumors. **(E)** GO enrichment analysis in hot tumors. **(F)** KEGG enrichment analysis in hot tumors.

To further explore the mechanisms of immune cell infiltration, we redefined cluster 1, cluster 2, and cluster 3 as cold tumors and cluster 4 as a hot tumor based on infiltration of immune cells and survival status. Hot tumors had longer OS and PFI than those in cold tumors (**Figures 5A, B**). Next, we analyzed the difference between the two groups at the transcriptional level. Hot and cold tumors showed different transcription patterns according to volcano plots (**Figure 5C**). In this study, 657 and 55 mRNAs were upregulated in hot and cold tumors, respectively. To further explore the function of DEGs, functional enrichment analyses were conducted using GO and KEGG. The GO analysis revealed that DEGs in cold tumors were significantly enriched in the “apical part of cell,” “actin-based cell projection,” and “apical plasma membrane” (**Figure 5D**), while those in hot tumors were primarily enriched in “T cell activation,” “regulation of lymphocyte activation,” “leukocyte cell–cell adhesion,” “regulation of T cell activation,” and “leukocyte proliferation” (**Figure 5E**). KEGG analyses of enrichment of DEGs revealed that DEGs in hot tumors were enriched mainly in “cytokine–cytokine receptor interaction,” “chemokine signaling pathway,” and “cell adhesion molecules,” which indicated an active immune response in hot tumors (**Figures 5F**); none of the KEGG annotations were enriched in cold tumors. These results suggested that the immune system was activated in hot tumors, especially the T cell-mediated immune response. Finally, the PPI networks revealed that the DEGs of hot tumors were mainly immune-related chemokines and cytokines, and DEGs of cold tumors were metabolic genes and Keratin family (**Supplementary Figures S5A, B**) .

### Inhibition of KRT23 Expression Promotes Infiltration of CD8^+^ T Cells

**Figure 6:**
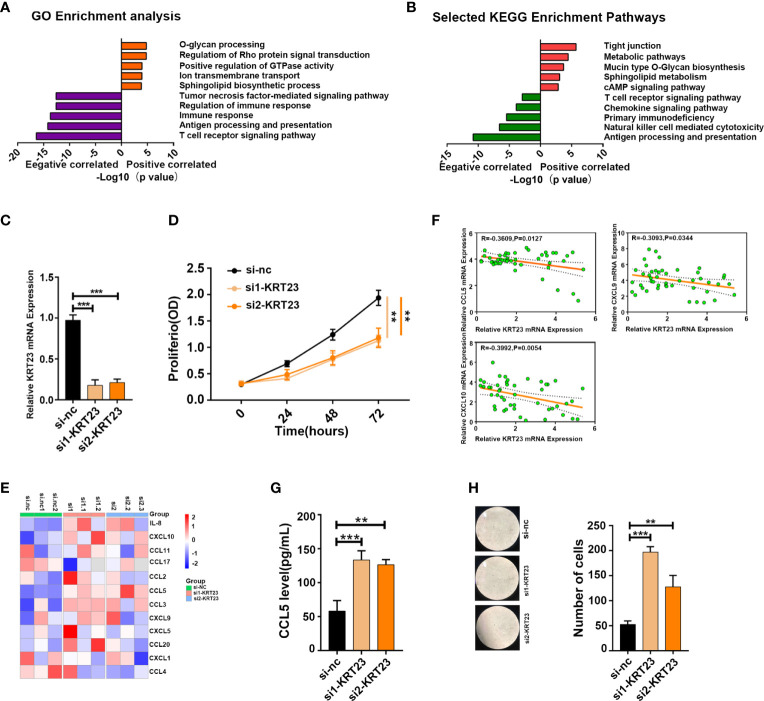
KRT23 promotes CD8^+^ T cell recruitment. **(A, B)** GO and KEGG analysis of KRT23-related genes. **(C)** qPCR analysis shows the knockdown efficacy of KRT23. **(D)** Proliferating rate of tumor cells with knockdown of KRT23. **(E)** The heatmap shows the concentration of cytokine and chemokine secreted by tumor cells with knockdown of KRT23. **(F)** Correlation of KRT23 and CCL5, CXCL9, and CXCL10 in tumor tissues of CC. **(G)** ELISA shows the CCL5 secretion by tumor cells with knockdown of KRT23. **(H)** Number of CD8^+^ T cells recruited by conditioned medium derived from tumor cells with knockdown of KRT23. **p ≤ 0.01, ***p ≤ 0.001.

The above results revealed a correlation between immune cell infiltration and longer survival, suggesting that inducing immune cell infiltration in cold tumors may enhance antitumor immunity and prolong survival time. Among the DEGs between cold and hot tumors, we found that KRT23 was most significantly expressed in cold tumors than in hot tumors. Pan-cancer analysis revealed that KRT23 has a higher expression in tumor tissues in most types of cancers (**Supplementary Figure S6A**) . Functional enrichment analyses using the KEGG and GO databases revealed that KRT23-related genes were negatively correlated with immune response (**Figures 6A, B**). Knockdown of KRT23 expression in HeLa cells inhibited cell proliferation (**Figures 6C, D**), which suggested an important role of KRT23. To explore how KRT23 affected immune cell infiltration, we used cytometric bead arrays to detect the chemokines derived from tumor cells with KRT23 knockdown. Results revealed that the secretion of CD8^+^ T cell-related chemokines (CCL5, CXCL9, and CXCL10) was increased in the knockdown group (**Figure 6E**). Then, we quantified the expression of KRT23 and CD8^+^ T cell-related chemokines in clinical tumor samples; the detailed information of patients was listed in **Table 1**. Results showed that KRT23 expression was negatively correlated with these chemokines, and this result was confirmed using TCGA database (**Figure 6F**; **Supplementary Figure S6B**) . We further found that KRT23 was negatively correlated with CD8^+^ T cells (**Supplementary Figure S6C**) . In addition, we validated the CCL5 expression because CCL5 changed most obviously after knocking down KRT23. Results revealed that the knockdown of KRT23 expression increased CCL5 secretion (**Figure 6G**). Transwell assays further indicated that knockdown of KRT23 promoted the recruitment of CD8^+^ T cells (**Figure 6H**).

**Table 1:** Clinicopathological parameters of patients with cervical cancer in our cohort in the study.

Characteristic	Number
**Histological type**	
Cervical squamous cell carcinoma	42
Non- squamous cell carcinoma	5
**History of neoadjuvant treatment**	
No	35
Yes	12
**Sample type**	
Primary	47
Metastatic	0
**Age at initial diagnosis**	
≥60	21
<60	26
**Clinical stage**	
I	9
II	22
III	11
IV	5
**HPV infection**	
Yes	19
No	2
NA	26
**Differentiation**	
Low	15
Moderate	28
High	4

### Construction and Validation of a Prediction Model Based on Differentially Expressed Genes

**Figure 7:**
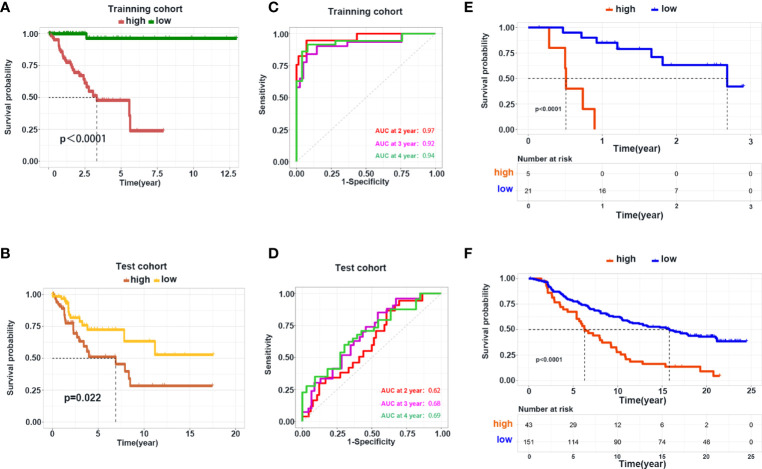
Construction and validation of the prediction model. **(A, B)** Kaplan–Meier curve shows the OS in the high- and low-risk group in the training and test cohorts. **(C, D)** ROC curve analysis shows the AUC of the prediction model in the training and test cohorts. **(E, F)** Validation of the prediction model using the dataset of patients with metastatic melanoma and urothelial cancer receiving immunotherapy treatment.

Next, we used DEGs to construct a prediction model. We performed a univariate Cox analysis followed by a Least absolute shrinkage and selection operator (LASSO) regression analysis (**Supplementary Figure S7A**) . To optimize the model, we carried out multivariate Cox analysis and finally identified 11 genes to construct our model (**Supplementary Figure S7B**) . Heatmaps were generated to reveal the expression of these genes in high- and low-risk groups in the training and internal test cohorts; the detailed information of patients was listed in **Table 2**. Survival analyses showed that patients with a high risk had shorter survival in the training and test cohorts (**Figures 7A, B**). To explore the accuracy of our model, we analyzed receiver operating characteristic (ROC) curves in the training and test cohorts at 1, 3, and 5 years. Our model yielded high area under the ROC curve (AUC) values (**Figures 7C, D**). Given that our model was established based on DEGs in hot and cold tumors, we hypothesized that this model could also predict tumor response to immunotherapy. Hence, we used two external cohorts of CC patients treated with immunotherapy. The results demonstrated that patients with a high risk had shorter survival in both cohorts (**Figures 7E, F**), suggesting that our model could predict the survival of patients who respond to immunotherapy.

**Table 2:** Clinicopathological parameters of patients with cervical cancer in TCGA dataset in the study.

Characteristic	Number
**Histological type**	
Cervical squamous cell carcinoma	235
Non- squamous cell carcinoma	50
**History of neoadjuvant treatment**	
No	285
Yes	0
**Sample type**	
Primary	283
Metastatic	2
**Age at initial diagnosis**	
≥60	227
<60	58
**Clinical stage**	
I	154
II	64
III	39
IV	22
NA	6
**Histologic grade**	
G1	17
G2	124
G3	116
G4	1
GX	27
**HPV infection**	
Yes	20
NA	265

## Discussion

Up to now, there is ample evidence suggesting that chemotherapy for CC is associated with limited efficacy. The optimal regimen against recurrent CC or mCC includes a combination of cisplatin, paclitaxel, and bevacizumab, associated with an overall response rate of 48% and a median survival of 17 months (20). Moreover, the side effects associated with radiotherapy limit their clinical application in CC (21), highlighting the need for new and efficient therapeutic strategies. In recent years, immunotherapy has demonstrated sustainable clinical response and is the first-line treatment for various tumors (22). “Cancer immunotherapy” is a general term that is described as harnessing a patient’s immune system to elicit antitumor effects (23). Antibodies against PD-1 and PD-L1 are commonly used for cancer immunotherapy. Their mechanism involved releasing the “inhibitory brakes” of T cells, resulting in robust activation of the antitumor immune response (24).

As previously stated, the major risk factor for CC is Human Papilloma Virus (HPV) infection (25), and the retained viral antigens in CC make immunotherapy an attractive option because they could be recognized as foreign. This rationale has led to the development of antibodies against PD-1 or PD-L1 assessed in several ongoing clinical trials (23, 26). Effective immunotherapy is contingent on the infiltration of lymphocytes and antigen-presenting cells. In general, the TME can be divided into two broad phenotypes: “T cell-inflamed” and “non-T cell-inflamed” (27). Several methods have been used to estimate the immune profile in the TME, encompassing ssGSEA, CIBERSORT, TIMER, MCP-counter, and xCell (28–32). ssGSEA and MCP-Counter use specific cell-maker genes and score the immune profile through the expression of these genes. CIBERSORT focuses on the ratios of each cell type using Nu-support vector regression. xCell integrates these methods and expands the cells that can be evaluated to 64 types. To more accurately reflect the level of immune cells in the TME of CC, we used two different methods. The comparison between tumor and adjacent normal tissues and correlation analysis of estimated immune cells yielded consistent findings, suggesting that these two methods can be used to estimate immune levels. We found that CC could be divided into 4 clusters based on consensus clustering, and clusters with higher immune infiltration yielded better survival rates. In a study by Wang et al. (33), CIBERSORT showed that CD4^+^ T cells represent an independent prognostic factor of CC. Meanwhile, immune cell infiltration has also been correlated with the response to chemotherapy (34).

In this study, we further redivided the 4 clusters into 2 subtypes: “hot” and “cold” tumors based on the immune levels. “Hot” tumors exhibited a T cell-inflamed phenotype, and “cold” tumors acted as a non-T cell-inflamed phenotype. Pathway enrichment analysis confirmed that “hot” tumors were associated with an active immune response. Cold tumors are characterized by the infiltration of few immune cells and are hence the most challenging to eradicate, accounting for their poor prognoses (35). Several strategies have been used to convert cold tumors to hot tumors: radiotherapy, chemotherapy, targeted therapy, and adoptive-cell therapy (36–40). In this study, we analyzed the differences between hot and cold tumors and identified *KRT23* as the most significantly upregulated gene in cold tumors. Keratin is the main component of epithelial cells, and malignant tumor cells originate from these epithelial cells. *KRT23* is a newly identified gene in the KRT family (41, 42). Studies have reported that KRT23 overexpression promotes the migration of ovarian cancer cells *via* epithelial–mesenchymal transition. Interestingly, KRT23 could promote the proliferation of colorectal tumor cells by increasing telomerase reverse transcriptase expression (43). Although the oncogenic role of KRT23 has been explored, it remains unclear how KRT23 affects the immune response. We found that KRT23 expression was negatively correlated with the immune response. Knockdown of KRT23 expression in tumor cells resulted in increased secretion of CCL5 and inhibited tumor cell proliferation. Our results corroborate that the inhibition of KRT23 expression enhances the antitumor response. Hence, a potential combination strategy of targeting KRT23 and immunotherapy could be a rational approach against CC.

The large difference in survival between hot and cold tumors inspired us to construct a prediction model based on the DEGs between the two types of CC tumors. This model performed well in the training cohort and internal and external validation cohorts. Hence, our model was reliable and could be used to guide clinical treatment. Over the years, several prediction models for CC have been documented in the literatures. Mei et al. (44) conducted immune profiling by ssGSEA and identified four immune-related prognostic gene signatures. Chen et al. (45) constructed a TME-related signature to predict the prognosis of CC. The results from those studies further substantiate our findings. Moreover, Ding et al. (46) screened survival-related immune genes and constructed a prediction model containing 13 genes. In addition, Yang et al. (47) constructed a prediction model based on ferroptosis-related genes. Of note, our model exhibited a high predictive power for the survival of patients with melanoma and urothelial cancer receiving immunotherapy.

However, there are some limitations in this study. First, we did not explore the effect of KRT23 on migration or apoptosis of tumor cells. Second, the prediction model lacks validation using clinical specimens.

## Conclusions

In the present study, we undertook a comprehensive analysis of the infiltration of immune cells in CC. We identified hot and cold tumors of CC; the former was associated with a more favorable outcome. Moreover, we demonstrated that KRT23 is a negative regulator of the immune response, and knockdown of KRT23 expression could promote CCL5 secretion. In addition, a prediction model based on DEGs between the two types of CC was established. This model performed well in predicting the survival of CC patients receiving immunotherapy. Overall, our findings provided novel insights into immune cell infiltration in CC and highlighted KRT23 as a potential target to enhance immunotherapy against CC.

## Data Availability Statement

The datasets used and/or analyzed during the current study are available from the corresponding author on reasonable request. The data that support the findings of this study are openly available on the online website UCSCXena (https://xenabrowser.net/). GSE77280 are available from the GEO database (https://www.ncbi.nlm.nih.gov/geo/), and data of patients with metastatic urothelial cancer treated with anti-PD-L1 agents were downloaded from the online website supplied in the article (http://research-pub.gene.com/IMvigor210CoreBiologies/).

## Ethics Statement

The studies involving human participants were reviewed and approved by the ethics committee of the First Affiliated Hospital of Zhengzhou University. The patients/participants provided their written informed consent to participate in this study.

## Author Contributions

XL designed the experiments, performed the experiments, and analyzed the data. YMC performed the experiments and analyzed the data. HS analyzed the data. YC designed the study and revised the article. All authors critically revised the article, approved the final version, and agreed to be accountable for all aspects of the article.

## Funding

This study was supported by the Henan Medical Science and Technology Project (Grant No.: LHGJ2090116).

## Conflict of Interest

The authors declare that the research was conducted in the absence of any commercial or financial relationships that could be construed as a potential conflict of interest.

The reviewer IB declared a shared parent affiliation with the authors to the handling editor at the time of review.

## Publisher’s Note

All claims expressed in this article are solely those of the authors and do not necessarily represent those of their affiliated organizations, or those of the publisher, the editors and the reviewers. Any product that may be evaluated in this article, or claim that may be made by its manufacturer, is not guaranteed or endorsed by the publisher.

## Supplementary Material

Supplementary Figure 1 the level of immune cells in tumor tissues that received radiotherapy or not. **(A-B)** Comparison of immune cells estimated by ssGSEA and xCell in tumor tissues with radiotherapy or not. ns: not significant, **p ≤* 0.05, ***p ≤* 0.01.

Supplementary Figure 2 Immune infiltration of 4 groups. **(A-B)** Infiltration of immune cells estimated by ssGSEA and xCell in 4 groups. **(C-D)** Expression of immune score, stromal score and tumor purity in 4 groups.*****p ≤* 0.0001.

Supplementary Figure 3 Immune profiles in 4 clusters.**(A)** Venn plot shows intersecting of samples in each cluster based on immune infiltration estimated by ssGSEA and xCell.**(B)**Expression profiles of antigen presentation-related genes in 4 clusters. **(C)**Expression profiles of chemokines in 4 clusters.**(D)**Expression profiles of checkpoints in 4 clusters.** *p ≤* 0.01, ****p ≤* 0.001, *****p ≤* 0.0001.

Supplementary Figure 4 Comparison of clinical parameters among the 4 clusters. **(A,B)** Bar charts shows the percentage of clinical stage and histologic grade among 4 clusters.

Supplementary Figure 5 PPI network of DEGs. **(A)** PPI netwok of genes upregulated in cold tumor. **(B)** PPI netwok of genes upregulated in hot tumor.

Supplementary Figure 6 Correlation of KRT23 and immune level. **(A)** Box plot shows the expression of KRT23 between tumor and normal tissue in pan-cancer dataset. **(B)** Correlation of KRT23 and CCL5,CXCL9 and CXCL10.**(C)** Correlation of KRT23 and immune cells.****p ≤* 0.001.

Supplementary Figure 7 Selection of genes for constructing prediction model. **(A)** Partial likelihood deviance plot. **(B)** Multi-Cox of selected genes by LASSO.**(C-D)** Heatmap showing gene expression of genes in prediction model in training and test cohort.
