# Supplementary figures and images for: Transcriptome Analysis Reveals the Immune Infiltration Profiles in Cervical Cancer and Identifies KRT23 as an Immunotherapeutic Target
Source: Front Oncol. 2022 Jun 24;12:779356. doi: 10.3389/fonc.2022.779356 (PMC9263098; doi:10.3389/fonc.2022.779356)

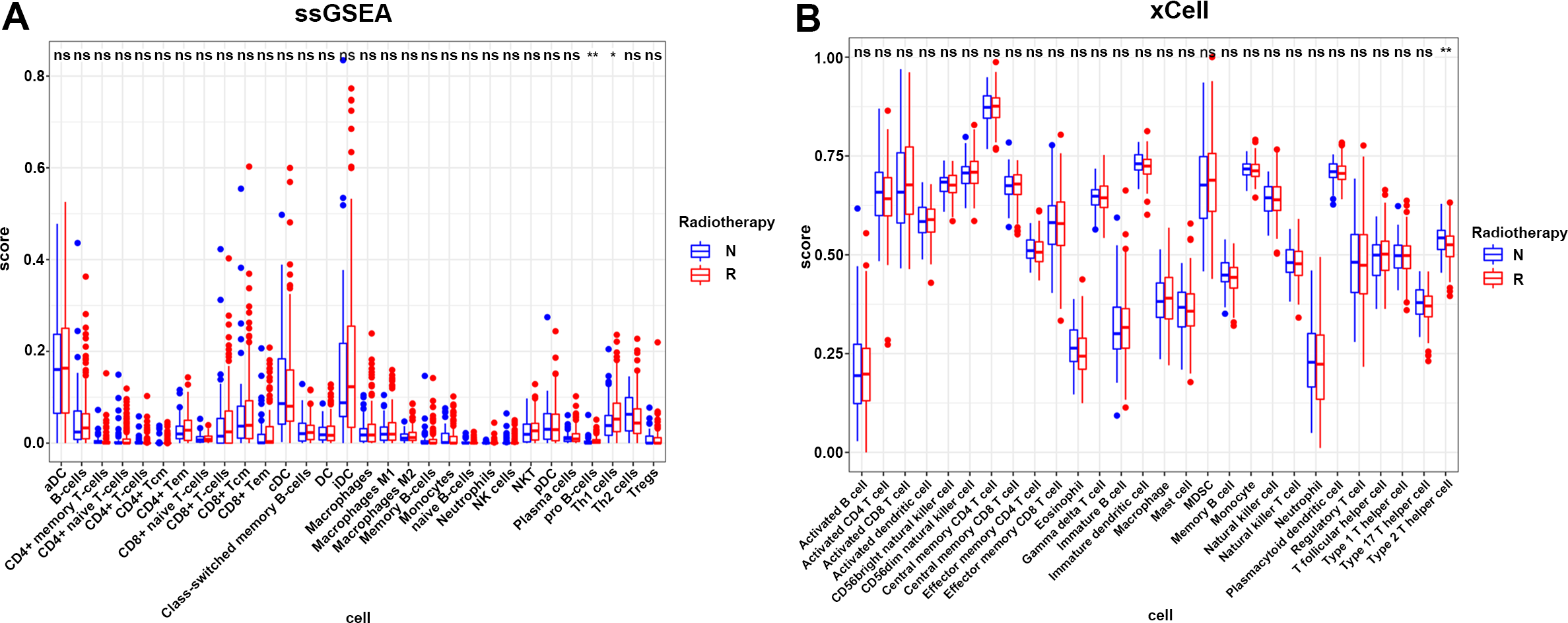

Supplement: Supplementary Figure 1 — the level of immune cells in tumor tissues that received radiotherapy or not. (A-B) Comparison of immune cells estimated by ssGSEA and xCell in tumor tissues with radiotherapy or not. ns: not significant, *p ≤ 0.05, **p ≤ 0.01. [file Image_1.tif]

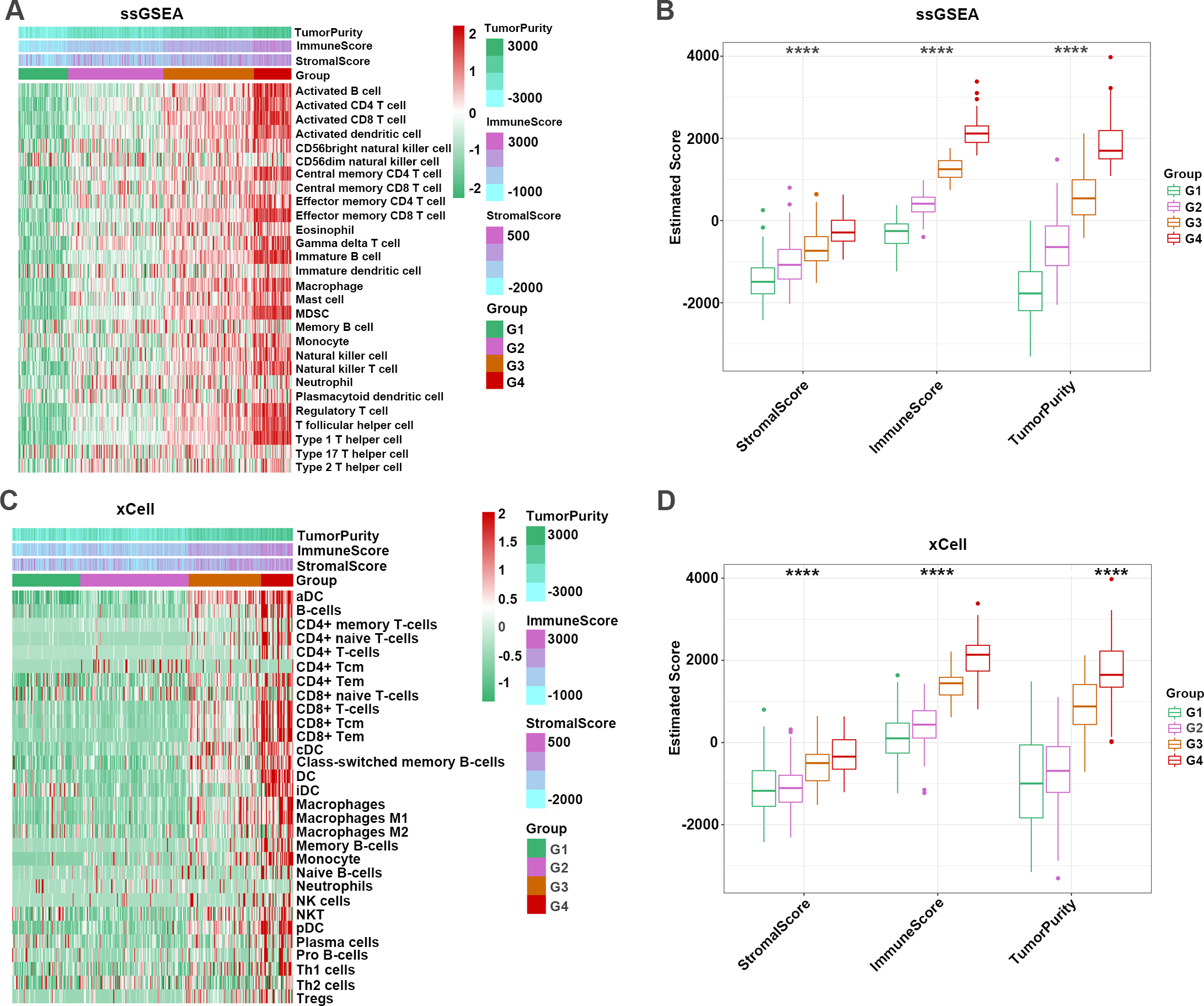

Supplement: Supplementary Figure 2 — Immune infiltration of 4 groups. (A-B) Infiltration of immune cells estimated by ssGSEA and xCell in 4 groups. (C-D) Expression of immune score, stromal score and tumor purity in 4 groups.****p ≤ 0.0001. [file Image_2.tif]

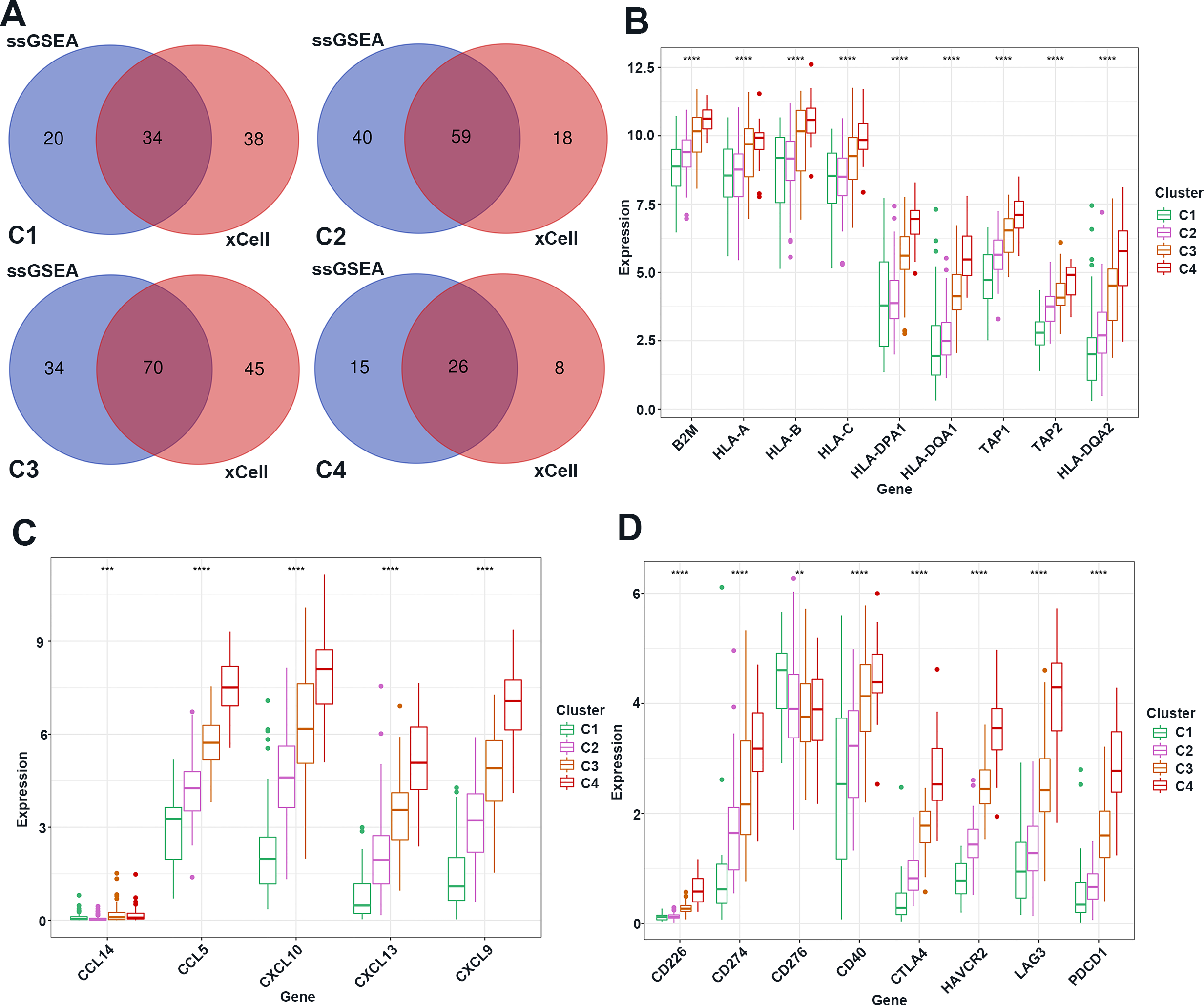

Supplement: Supplementary Figure 3 — Immune profiles in 4 clusters.(A) Venn plot shows intersecting of samples in each cluster based on immune infiltration estimated by ssGSEA and xCell.(B)Expression profiles of antigen presentation-related genes in 4 clusters. (C)Expression profiles of chemokines in 4 clusters.(D)Expression profiles of checkpoints in 4 clusters.** p ≤ 0.01, ***p ≤ 0.001, ****p ≤ 0.0001. [file Image_3.tif]

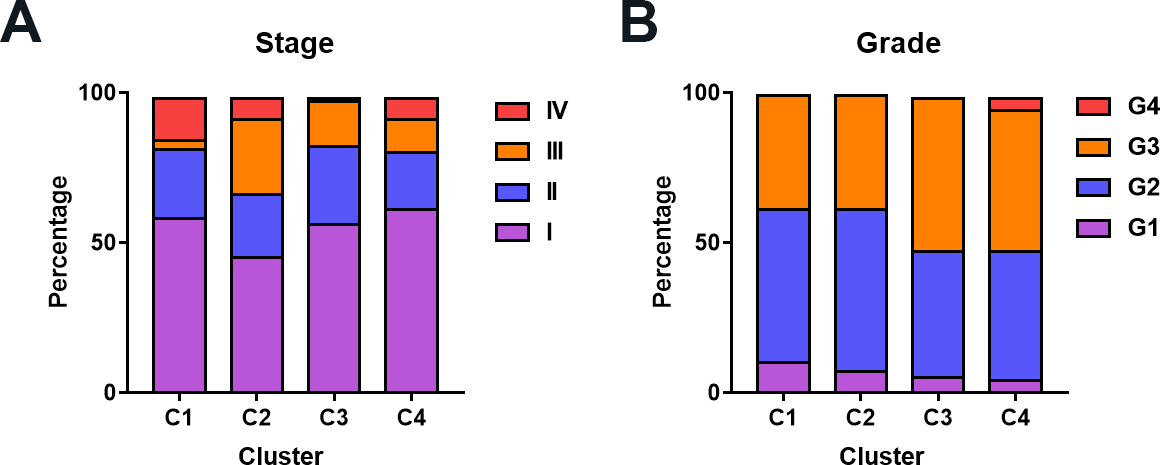

Supplement: Supplementary Figure 4 — Comparison of clinical parameters among the 4 clusters. (A,B) Bar charts shows the percentage of clinical stage and histologic grade among 4 clusters. [file Image_4.tif]

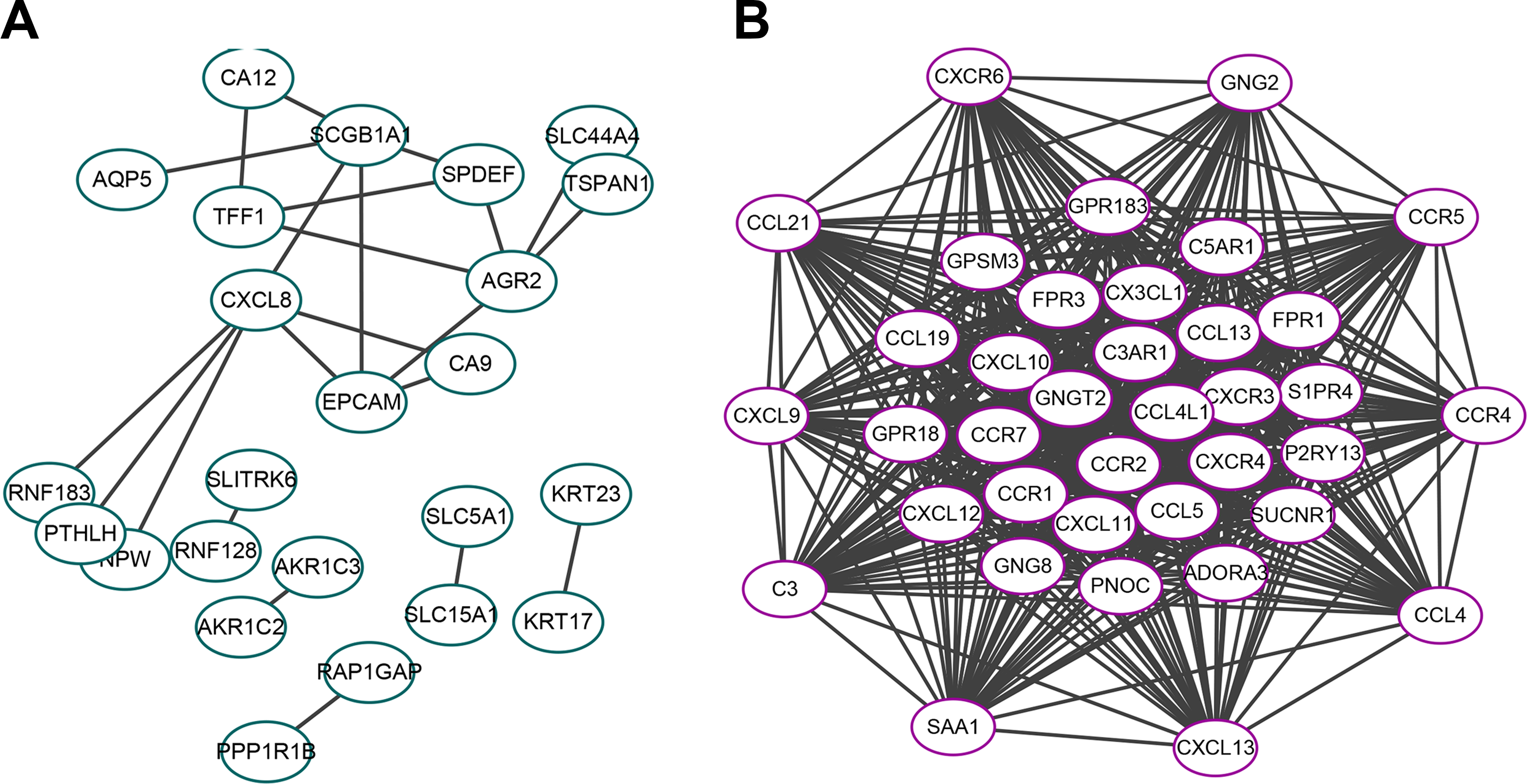

Supplement: Supplementary Figure 5 — PPI network of DEGs. (A) PPI netwok of genes upregulated in cold tumor. (B) PPI netwok of genes upregulated in hot tumor. [file Image_5.tif]

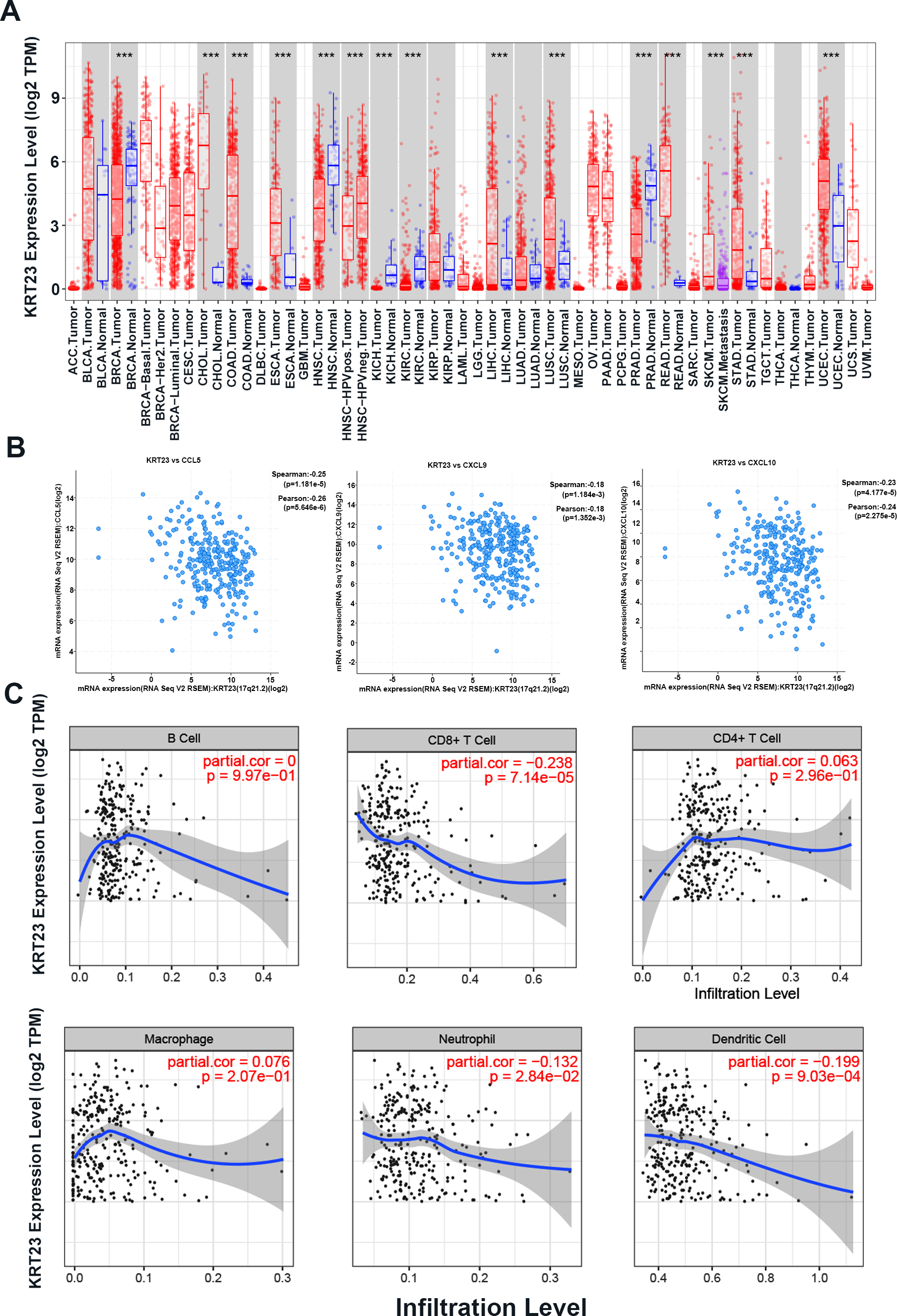

Supplement: Supplementary Figure 6 — Correlation of KRT23 and immune level. (A) Box plot shows the expression of KRT23 between tumor and normal tissue in pan-cancer dataset. (B) Correlation of KRT23 and CCL5,CXCL9 and CXCL10.(C) Correlation of KRT23 and immune cells.***p ≤ 0.001. [file Image_6.tif]

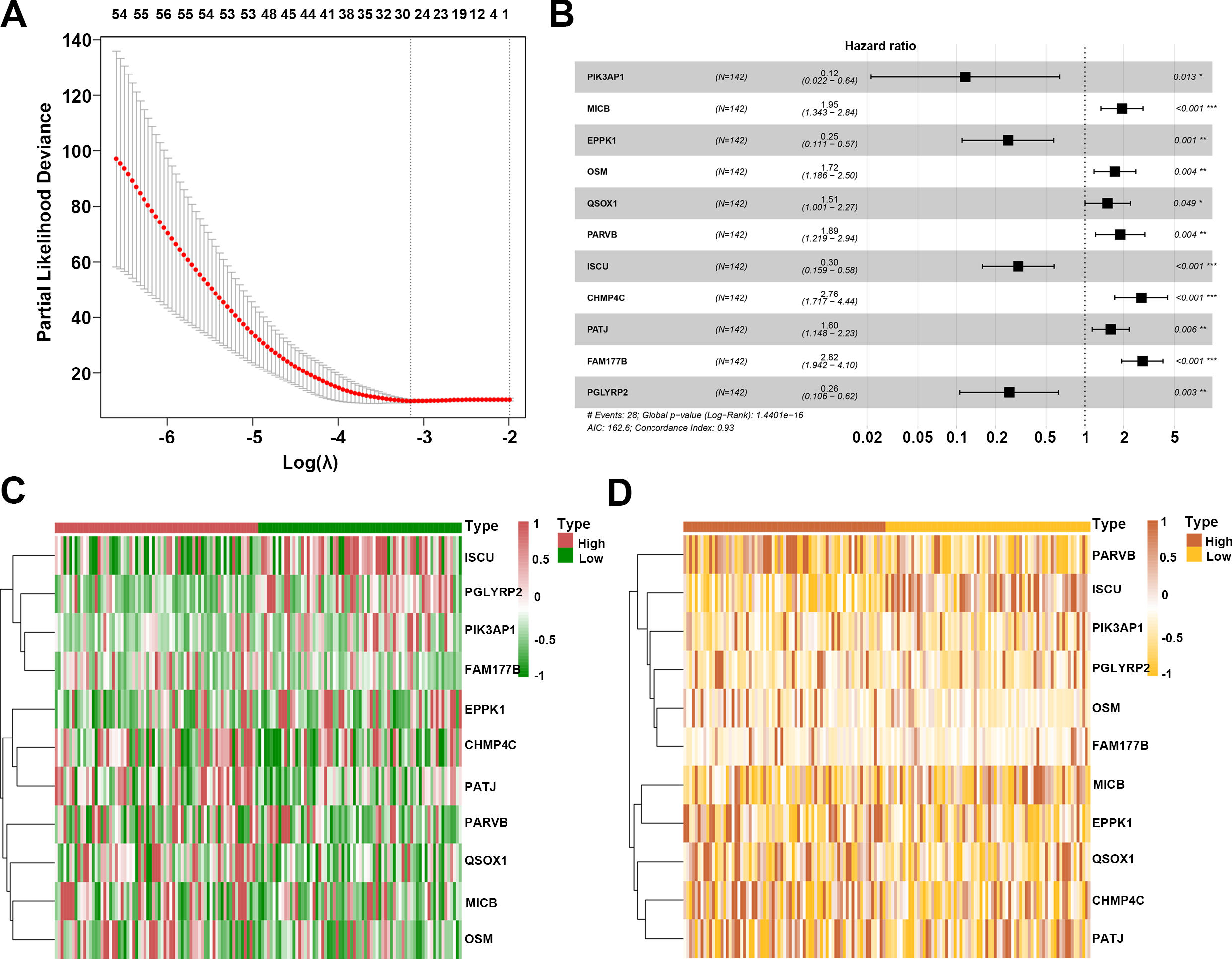

Supplement: Supplementary Figure 7 — Selection of genes for constructing prediction model. (A) Partial likelihood deviance plot. (B) Multi-Cox of selected genes by LASSO.(C-D) Heatmap showing gene expression of genes in prediction model in training and test cohort. [file Image_7.tif]
