# Supplementary material for: Transcriptome Analysis Reveals the Immune Infiltration Profiles in Cervical Cancer and Identifies KRT23 as an Immunotherapeutic Target
Source: Front Oncol. 2022 Jun 24;12:779356. doi: 10.3389/fonc.2022.779356 (PMC9263098; doi:10.3389/fonc.2022.779356)
Supplement: Supplementary file 8 [file Table_1.docx]

| Gene | forward | reverse |
| --- | --- | --- |
| KRT23 | TACTAGGCGGAAATGGGAAGG | TCTTACCATCCACTATCTGCTCC |
| CXCL9 | CCAGTAGTGAGAAAGGGTCGC | AGGGCTTGGGGCAAATTGTT |
| CXCL10 | GTGGCATTCAAGGAGTACCTC | TGATGGCCTTCGATTCTGGATT |
| CCL5 | CCAGCAGTCGTCTTTGTCAC | CTCTGGGTTGGCACACACTT |

Supplementary Table1: Primers used in this study

Supplementary Table2: the sequence of KRT23 siRNA

| SiRNA-KRT23 |  |  |
| --- | --- | --- |
| Si-1 | AUGAGAAUGAGCUCUUUCCUC | GGAAAGAGCUCAUUCUCAUGA |
| Si-2 | GGAGGAUAUGAGACAAGAAUA | UUCUUGUCUCAUAUCCUCCAG |
